# Detection and Characterization of Cancer Cells and Pathogenic Bacteria Using Aptamer-Based Nano-Conjugates

**DOI:** 10.3390/s141018302

**Published:** 2014-09-29

**Authors:** Vinayakumar Gedi, Young-Pil Kim

**Affiliations:** 1 Department of Life Science, Hanyang University, Seoul 133-791, Korea; E-Mail: vinayreddyg@live.com; 2 Research Institute for Natural Sciences, Hanyang University, Seoul 133-791, Korea; 3 Institute of Nano Science and Technology, Hanyang University, Seoul 133-791, Korea

**Keywords:** aptamer, cell-SELEX, bacteria, cancer cell, nanoparticle, nanomaterial

## Abstract

Detection and characterization of cells using aptamers and aptamer-conjugated nanoprobes has evolved a great deal over the past few decades. This evolution has been driven by the easy selection of aptamers via *in vitro* cell-SELEX, permitting sensitive discrimination between target and normal cells, which includes pathogenic prokaryotic and cancerous eukaryotic cells. Additionally, when the aptamer-based strategies are used in conjunction with nanomaterials, there is the potential for cell targeting and therapeutic effects with improved specificity and sensitivity. Here we review recent advances in aptamer-based nano-conjugates and their applications for detecting cancer cells and pathogenic bacteria. The multidisciplinary research utilized in this field will play an increasingly significant role in clinical medicine and drug discovery.

## 1. Introduction

Extensive genetic information on a large panel of diseases has been necessarily accompanied by targeting cells, which is of great significance for early diagnosis and effective therapy. While traditional methods used to identify the differences between normal and abnormal cells or between host and pathogenic cells rely upon the long-standing observation of cell phenotypes or PCR-based molecular diagnosis [1–3], straightforward methods to target aberrant cells have been developed using sensing molecules, such as monoclonal antibodies, smaller antibody fragments, peptides and low-molecular mass ligands [4–7].

As single-stranded nucleic acids (ssDNA or RNA), aptamers offer several advantages over other sensing molecules for diagnostic and therapeutic applications [8]. In addition to being chemically stable, cost-effective and producible on a large scale, aptamers also possess an intermediate size (between antibodies and small peptides) and have comparable or higher binding affinities for their targets. These targets range from small molecules to whole cells [9–11] via an *in vitro* selection process known as systematic evolution of ligands by exponential enrichment (SELEX) [12]. In particular, the use of aptamers that utilize cell-SELEX have contributed to significant advances in disease diagnosis and drug development on the cellular and tissue levels [13,14] and are superior to antibody-based diagnostic and therapeutic applications. Although there is no prior knowledge of specific targets, a counter-selection strategy using control cells (negative-SELEX) with target cell-SELEX gives rise to cell-specific aptamers with high stability and binding affinity. These allow for the reliable identification of targeted cells at the molecular level in combination with various analysis tools, such as fluorescence-activated cell sorting or the enzyme-linked immunosorbent assay [15,16]. Unlike antibodies based on purified receptors, aptamers are more attainable and responsive in living cells because they are selected from intact cells; it is not necessary to consider the conformational changes of the recognition domains in the cell membrane. Among many other types of cells, bacteria and tumor cells have been of primary interest [17,18], due to their involvement in many human diseases.

To exploit the full potential of aptamer-based cell targeting, aptamers can be combined with nanomaterials, such as gold nanoparticles (AuNPs), silica NPs (SiNP), graphenes, magnetic NPs (MNP) and quantum dots (QDs). This aptamer-nanomaterial hybridization process is easily accomplished due to the simple chemical modification and well-defined structures of aptamers [19,20]. These hybrid materials are expected to improve target diagnosis and therapy with higher sensitivity and selectivity compared to aptamer-only-based targeting strategies. Most importantly, due to the large surface area, multivalent structure, and relevant physiochemical properties of nanomaterials, aptamer-nanomaterial hybrids should provide signal amplification and an increased target binding affinity in a multivalent manner.

Although many reviews discuss aptamer-functionalized nanomaterials [21–24], this review includes recent advances in isolation, detection, and characterization of cells using the aptamer-nanomaterial hybrid systems via cell-SELEX. Furthermore, to avoid redundancy with other recent reports, which provide broad insight into aptamers and their recent applications in cancer diagnosis and therapy [25], we focus on applications based on living bacteria and cancer cells with recent advances in targeting strategies.

## 2. Cell-SELEX

**Figure 1.**
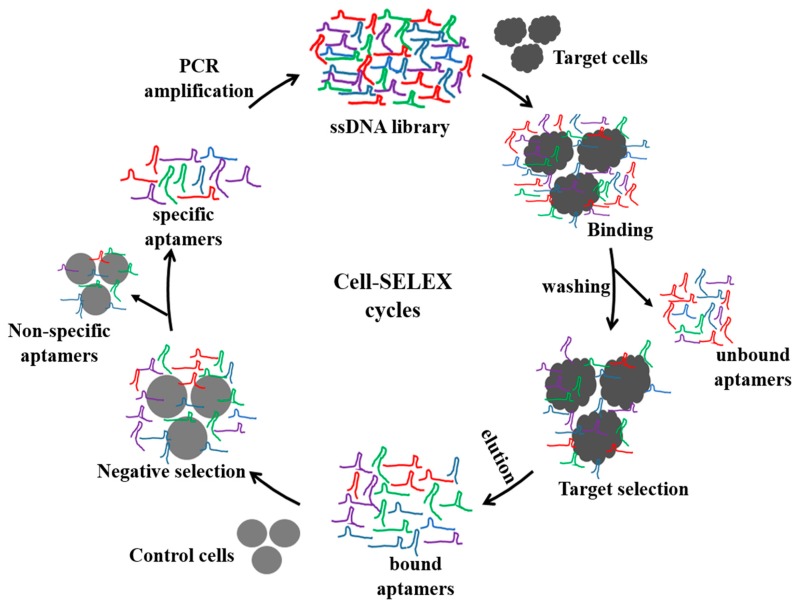
Schematic representation of cell-SELEX.

Among living cells, aptamers have been developed to detect whole single cells, where they bind to cell surface protein targets. As summarized in Tables 1 and 2, recent reports of cell-SELEX have mainly focused on specific aptamers against various cancer cells [15,16,26–36] and whole bacteria [18,37–50]. As depicted in Figure 1, to generate a specific aptamer, a random ssDNA/RNA aptamer library is initially incubated with target cells for a specific period of time (from 30 min to 1 h) to allow for target-specific recognition. This is followed by centrifugation to remove the unbound aptamer. The cells are then washed several times, and the aptamers bound to the surfaces of the cells are eluted by heat-induced denaturation. The eluted aptamers are amplified and further subjected to negative selection using control cells; negative selection plays an important role in cell-SELEX to remove aptamers binding to common cell surface proteins. In order to increase the efficiency of negative selection, the concentrations of the control cells are often in five- to ten-fold excesses compared to the target cells. This selection can be performed for several rounds until the produced aptamers have a high binding affinity (nanomolar range K_d_ values) toward the target cells. Consequently, the aptamers that are evolved from cell-SELEX are implemented for detection of cancer or infectious cells, discovery of new biomarkers, and disease therapy [10,13,14,51–53]. Furthermore, cell-binding aptamers are suitable for the development of *in vivo* tumor targeting [54,55], receptor-dependent neutralization [56–58], and drug delivery systems [59,60].

## 3. Aptamer-Conjugated Nanomaterials in Whole-Cell Detection

The hybridization of aptamers with nanomaterials is of particular interest due to the unique properties and utilities of nanomaterials, including their small size, increased surface-to-volume ratio, and a wide range of sensing modules, that include metal and semiconductor core materials. To date, nanomaterials can be easily fashioned with aptamers via direct covalent linkage or by non-covalent interactions. This modification aim to address several shortcomings caused by less than ideal detection sensitivity, assay time, and target specificity [22,61–64]. While several strategies have been reported for conjugating aptamers with nanomaterials [19], nanomaterials are generally used as either supporting reservoirs for immobilizing ligands or as labeling agents for signal amplification. Importantly, aptamer-conjugated-nanoparticles (Apt-NP) are capable of being detected by optical, electrochemical, fluorescence or mass-sensitive analytical techniques, depending on their physical and/or chemical properties. Compared with antibody-based cell assays, aptamers screened from cell-SELEX provide much more versatile strategies for recognizing cells because the smaller size of the aptamers is beneficial for designing nano-hybrid sensors in a highly compact regime, which contributes to the signal amplification effect and increases target binding affinity. Since the aptamers are also directly selected from living cells, Apt-NPs would be more effective for targeting cells, compared to antibodies or their nano-hybrid formats, which often do not reflect the conformational changes of receptor proteins in living cells. In addition, nanomaterials could protect aptamers from being digested by nucleases, indicating that Apt-NPs can act as stealthy carriers for drug or gene delivery. Here we provide an overview of the nanomaterials that are widely used in aptamer-based sensors for detecting cancer cells and pathogenic bacteria via limit of detection (LOD).

### 3.1. Aptamer-Conjugated AuNPs

AuNPs are widely used in bioanalysis due to their simple synthesis and unique spectral properties. AuNPs have typically been produced from the chemical reduction of AuCl_4_ ions by agents, such as citric acid, and their size can be easily controlled [65–67]. Due to their biocompatibility and optical properties triggered by the surface plasmon phenomenon, AuNPs conjugated with aptamers from cell-SELEX have been extensively utilized in the fields of cellular diagnostics and imaging. The distance-dependent fluorescence quenching behavior of AuNPs enables distinct biomolecular interactions in close proximity, which is limited to cellular assays, due to the difficulty in target-labeling and the large amount of noise caused by interferants. In contrast, AuNP-based plasmonic assays provide a simpler method to amplify the signal; therefore, plasmon assays prevail in cellular analysis and are utilized depending on the change in localized surface plasmon resonance (LSPR) or enhancement caused by AuNP-triggered self-assembly or AuNP-conjugated probes, respectively [68–72].

Taking advantage of these optical properties, Medley *et al.* developed a simple colorimetric assay for cancer cells using aptamer-conjugated-AuNPs (Apt-AuNP) [73]. A thiol-modified aptamer which was specifically obtained using cell-SELEX for CCRF-CEM acute leukemia cells [15], was conjugated with AuNPs and targeted to assemble on the surfaces of cells through the target recognition ability of the aptamer. The binding and assembly of Apt-AuNPs on the cell surfaces bring AuNPs into close proximity with one another, causing a shift in the extinction spectra, which can then be used to quantify the number of cells [73]. In a similar study, Liu *et al.* detected Ramos cells using AuNPs and a pair of previously identified aptamers from cell-SELEX [29,74]. Briefly, a sample containing Ramos cells and Apt-AuNPs was applied to a secondary aptamer immobilized on a strip surface. Ramos cells interacted with Apt-AuNPs and migrated over the strip to the region containing the surface-immobilized secondary aptamers. The accumulation of Apt-AuNPs was then visualized as a red color and quantified. With this technique, as few as 4000 Ramos cells could be detected with the naked eye; this sensitivity increased to 800 cells when using a portable strip reader [74]. Wu *et al.* also demonstrated the salt-induced color change of AuNPs for the detection of *E. coli* and *Salmonella typhimurium* [75]. They incubated Apt-AuNPs with the target bacterial cells for 10 min. These were then aggregated upon the addition of NaCl. The subsequent color change from red to purple was simply detected either visually or with UV-vis spectroscopy [76]. This colorimetric method was able to detect 105 CFU·mL^−1^ of *E. coli* or *S. typhimurium* [75] without requiring any expensive instrumentation or labeling process.

**Figure 2.**
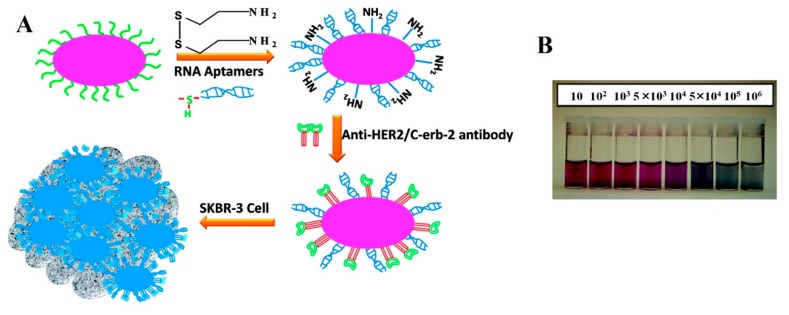
(**A**) Schematics of the conjugation of an aptamer and monoclonal anti-HER2 antibody with oval-shaped AuNPs and their accumulation on a cell surface; (**B**) The effect of SK-BR-3 cell concentration on the colorimetric change (pink to bluish) of oval-shaped AuNPs. The image was adopted from [77].

Recently, Lu *et al.* reported oval-shaped AuNPs conjugated with an aptamer identified through cell-SELEX [30] and a monoclonal anti-HER2/c-erb-2 antibody for the sensitive and selective detection of breast cancer SK-BR-3 cells, respectively [77]. As shown in Figure 2, a colorimetric change was observed from pink to bluish, as AuNPs accumulated on the surfaces of SK-BR-3 cells. The LOD of the colorimetric assay was 10^4^ cells·mL^−1^. The sensitivity was further improved by employing a two-photon scattering technique (TPS), which was powerful enough to detect the small changes in the sizes of NPs, leading to a LOD improved by as much as two orders of magnitude (100 cells·mL^−1^) compared to the simple colorimetric assay [77]. In comparison, the TPS intensity was two-fold higher when the cells were incubated with the AuNPs conjugated with both aptamer and antibody compared to the AuNPs conjugated with either aptamer or antibody. The conjugation of anti-HER2/c-erb-2 antibody together with the aptamer clearly detected the various breast cancer cells, which depended on the overexpression levels of HER2 [77]. In another approach, Chang *et al.* measured the resonance light scattering of Apt-AuNPs for the detection *Staphylococcus aureus* cells [44]. They employed ssDNA-modified AuNPs, where the ssDNA acted as an adaptor sequence for aptamer hybridization. The Apt-AuNPs were then bound with *S. aureus* cells and subjected to resonance light-scattering analysis using a laser light source, objective lens, photodiode and digital voltmeter after being eluted with NaOH. The result showed that the sensitive resonance light-scattering analysis was able to detect as few as 312 cells [44].

Improved detection sensitivity at lower target concentrations was accomplished using a signal amplification method in which silver ions are reduced on the AuNP surface by a reductant (e.g., hydrazine). By employing two different target ligands consisting of the antibody and the aptamer, HER2-overexpressing SK-BR-3 breast cancer cells were initially captured by monoclonal anti-HER2 antibodies immobilized onto a nanocomposite, comprised of self-assembled AuNPs [78]. The bound target cells were further conjugated with hydrazine-AuNP-aptamer (Hyd-AuNP-Apt) which was also specific to HER2. The signal transduction was performed via silver nitrate addition, which was selectively reduced to silver metal by hydrazine and specifically deposited onto the Hyd-AuNP-Apt. Therefore, the deposited silver metal was analyzed using square wave stripping voltammetry to determine the amount of HER2-overexpressing cells. Under optimized conditions, the LOD of SK-BR-3 breast cancer cells is 26 cells·mL^−1^ [78].

A similar strategy with a few modifications, has also been reported to detect *S. typhimurium* [79]. A biotinylated aptamer immobilized on an avidin-coated microplate was used to capture the target bacteria, the bound bacteria were further detected by secondary Apt-AuNPs, which was followed by the addition of a silver enhancer solution. The combination of Apt-AuNPs and the silver staining method had a detection limit as low as 7 CFU·mL^−1^ [79].

Yi *et al.* developed a different electrochemical method for detecting Ramos cancer cells using Apt-AuNPs and enzyme-triggered silver enhancement [80]. They immobilized a thiol-modified aptamer onto the Au electrode to capture the Ramos cells. As a detection probe, a biotinylated secondary aptamer was subsequently employed to amplify target cells with the addition of streptavidin-alkaline phosphatase (ALP); the presence of ALP on the surface promoted enzymatic silver ion reduction and deposition onto the Au-electrode, allowing for simple electrochemical detection. Through two aptamer-based sandwich assays and enzymatic reaction, the LOD of Ramos cells was determined to be as low as 10 cells [80].

### 3.2. Aptamer-Conjugated Magnetic Nanoparticles

Magnetic nanoparticles (MNPs) are composed of inorganic nanocrystals with metals, metal alloys, and metal oxides as their magnetic cores [81]. Among MNPs, superparamagnetic iron oxide NPs (SPIONs), including Fe_3_O_4_ and g-Fe_2_O_3_ have been a major research focus [82] due to their reversibly switched response to an external magnetic force. Importantly, the surfaces of MNPs can be modified with other metal atomic layers, such as Au, Ag, and Al_2_O_3_, which serve as effective conjugating sites for various ligands and labeling groups (e.g., aptamers, proteins and fluorescent dyes) [83,84]. When combined with cell-specific aptamers, multifunctional MNPs have additional advantages in terms of their ability to detect living cells compared to other nanoparticles conjugated with aptamers. Magnetic control without centrifugation enables aptamer-conjugated-MNPs (Apt-MNP) to be extensively applied to the non-invasive separation, extraction, and enrichment of target cells. This is also relevant to increasing the detection sensitivity by enriching low-abundant targets from complicated samples. In addition, MNPs can act as both a nanostructured active site with large surface area and as an additional signal generator when they are combined with other detecting molecules, such as fluorophores, enzymes, or other metals.

**Figure 3.**
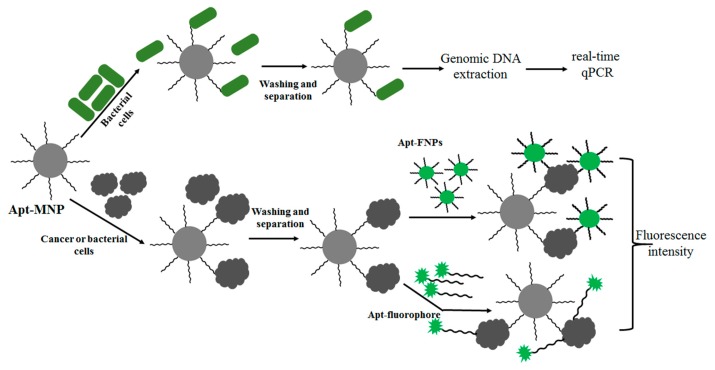
General strategies for cancer and bacterial cell detection using Apt-MNPs. In general, the Apt-MNPs are used to separate and capture the target cells and then detected using additional signal amplification methods. The captured cancer/bacteria cells are generally detected using either Apt-fluorophores or Apt-FNPs. For bacteria, genomic DNA based real-time qPCR was successfully applied for sensitive detection.

A recent report demonstrated this principle: Apt-MNPs were exploited to rapidly and easily capture and concentrate bacterial cells (Figure 3). The captured bacterial cells were identified with high sensitivity using subsequent detection methods, including fluorophore-conjugated aptamers [46] and real-time qPCR [43,85]. As a result, this strategy was shown to be useful for detecting various bacteria such as *S. typhimurium*, *Listeria* and *E. coli* [43,46,85]. In order to obtain a rapid and efficient method for targeting cancer cells, Tan and colleagues used a similar method for the detection of CCRF-CEM acute leukemia cells using modified Apt-MNPs (silica-coated iron oxide MNPs modified with avidin) and Apt-FNPs (Rubpy-doped silica NPs) [86]. They also demonstrated that it was possible to perform multiple cell type extraction from a complex mixture using Apt-MNPs and Apt-FNPs [87]. As a proof of concept, three different Apt-MNPs and three different dye doped Apt-FNPs (Cy5, Rubpy and TMR), specific to three different cells (Ramos, Toledo and CEM cells), were used for separation and detection, respectively.

The conjugated aptamers were individually obtained through whole cell-SELX and are known for their specific binding towards target cell [15,29]. The cells bound to the respective Apt-MNPs were validated by flow cytometer analysis with the dye-doped Apt-FNPs after magnetic separation. Surprisingly, the combination of two different Apt-NPs resulted in an LOD with very low detection sensitivity (250 cells). Another experiment by the same group showed that among different MNP sizes, 60-nm particles resulted in the greatest measurable intensity with an LOD of 152 cells. Interestingly, the detection sensitivity against the same target cells was further improved by conjugating the MNPs with multiple aptamers, such as two-, three- and four-Apt-MNPs, leading to an LOD as low as 45, 97 and 6.6 cells, respectively [88]. Tan's group also utilized the multiple aptamer-MNPs to detect CCRF-CEM cells by measuring the change in spin-spin relaxation time (ΔT2) [89]. This assay method was very effective and could detect as few as 10 cells. They suggested that this method would be suitable for differentiating various cell types through arrayed-type pattern recognition based on the specific signature between the target cells and the Apt-MNPs.

**Figure 4.**
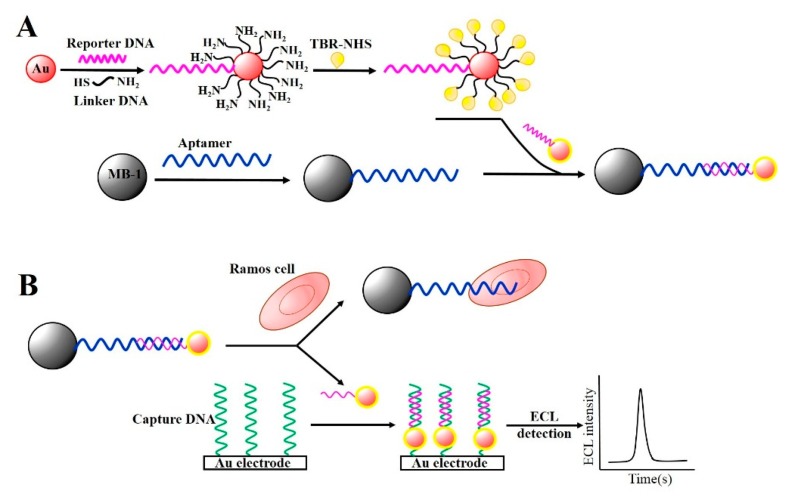
(**A**) Schematics of the conjugation of AuNPs with TBR and a linker DNA that partly hybridizes with the aptamer to create a signal amplification molecule. The linker DNA is then hybridized with the Apt-MBs to form a bio-complex: AuNP-Apt-MB; (**B**) Illustration of ECL-based detection of cancer cells. In the presence of target cells, the AuNPs are released from the Apt-MBs and hybridized with the capture DNA that has been modified on the Au electrode, which is then detected using electrochemiluminescence. The image was adopted from [90].

Other attempts have been made utilizing aptamer-conjugated magnetic beads (Apt-MBs) in combination with AuNPs for signal amplification because AuNPs are responsible for generating electrochemical or electrochemiluminescence (ECL) signals. Ding *et al.* reported the construction of a magnetic biocomplex consisting of Apt-MBs and reporter DNA-AuNPs, where the AuNPs were modified with a signal DNA labeled with tris(2,2′-bipyridyl)ruthenium(II) (TBR) and a linker DNA that can partially hybridize with the aptamers. This hybrid complex was referred to as AuNP-Apt-MB (Figure 4A) [90].

When the AuNP-Apt-MB was applied to detect Ramos cells, the DNA-linked AuNP was used as a single reporter. In the presence of target cells in the biocomplex, the complexes of AuNP-Apt-MBs were separated because the target cells were bound to the aptamer on the MBs. This released the DNA-AuNPs due to the loss of binding sites on the MBs. Consequently, the reporter DNA in the Apt-AuNPs was hybridized with the capture DNA on the Au-electrode, contributing to the ECL generation via the TBR loaded on the AuNPs (Figure 4B). They showed that the ECL intensity of the TBR was directly proportional to the amount of Ramos cells in the biocomplex, and the LOD was determined to be 50 cells·mL^−1^ under optimal conditions. They also examined whether magnetic nanocomposites, as opposed to micro-sized magnetic beads, showed better efficiency for capturing target cells. As a result, magnetic nanocomposites provided easier separation and increased ECL signals [91], lowering the LOD to five cells·mL^−1^. Zang *et al.* demonstrated a similar strategy to extract HL-60 cancer cells using Apt-MBs and AuNP signal amplification [92]. However, instead of TBR, they used a CdS nanocluster film to provide the ECL signal. This strategy was well-suited for the detection of different cancer cells at concentrations as low as 20 cells·mL^−1^.

Zhang *et al.* demonstrated the electrochemical detection of CCRF-CEM acute leukemia cells using the Fe_3_O_4_ MNPs and AuNP-catalyzed silver deposition enhancement [93]. Due to their large surface-to-volume ratio, the Apt-MNPs were used as carriers and reservoirs for loading a large amount of AuNPs. A competitive binding assay in the presence of target cells enabled the Apt-MNP-AuNP complexes to be separated by the magnetic support. The released AuNPs were subsequently treated via silver deposition. The AuNP-catalyzed silver deposition enhancement showed high sensitivity with an LOD of 10 cells·mL^−1^ [93].

### 3.3. Aptamer-Conjugated Silica Nanoparticles

Silica nanoparticles (SiNPs) have emerged as promising candidates for characterizing cells due to their excellent biocompatibility, easy separation, broad size range (5−1000 nm) with large surface area, and superb carrier ability with versatile labeling techniques [94]. When SiNPs were conjugated with aptamers (Apt-SiNPs), target analytes were easily separated from the biocomplex through simple centrifugation [95]. The large surface area of SiNPs also allowed for the encapsulation of a large number of fluorophores in order to generate intense fluorescence [96], enabling them to function as a signal enhancer for the detection of cancer cells. For example, Wang *et al.* developed fluorescence resonance energy transfer (FRET)-based SiNPs doped with three different dyes that exhibit multiple colors at one wavelength [97]. These SiNPs exhibited excellent fluorescence intensity with weak photobleaching properties when targeting CEM cancer cells after being conjugated with aptamers [97].

**Figure 5.**
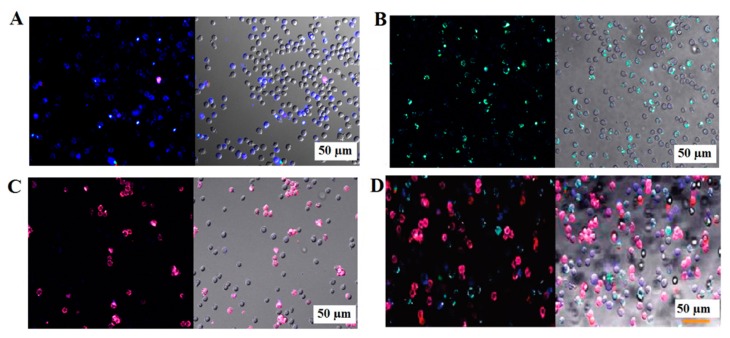
Confocal microscopic images showing a mixture of three cells (Toledo, CEM, and Ramos) incubated with (**A**) FAM-T1-SiNP; (**B**) FAM-R6G-sgc8-SiNP; and (**C**) FAM-R6G-ROX-TD05-SiNP (specific for Toledo, CEM and Ramos cells, respectively); (**D**) Mixture of the three cells incubated with all three different Apt-dye-SiNPs. The aptamers T1, sgc8 and TD05 are known to specifically bind with Toledo, CEM and Ramos cells, respectively. The image was adopted from [99].

Tan and his colleagues demonstrated FRET-based SiNPs labeled with several fluorophores and aptamers, and exhibited the feasibility of multiplexed detection of cancer cells [98,99]. Three different SiNPs contained a single dye (FAM), two dyes (FAM and R6G), and three dyes (FAM, R6G and ROX) that were conjugated with aptamers (FAM-T1-SiNP, FAM-R6G-sgc8-SiNP, FAM-R6G-ROX-TD05-SiNP for Toledo, CEM and Ramos cells, respectively) via neutravidin-biotin interaction [98]. These SiNPs improved the specificity of the as-selected aptamers against the corresponding cell types with a nanomolar range in the binding affinity [99]. As shown in Figure 5, three different fluorophore/aptamer-conjugated SiNPs specifically bound to their corresponding target cells in a three-cell mixture, as evidenced by the distinct colors of the attached SiNPs. Recently, Cai *et al.* synthesized Rubpy-doped SiNPs for MCF-7 cell imaging using a MUC-1 specific aptamer, showing that these Apt-SiNPs have better photostability and selectivity than the dye-labeled MUC-1 aptamer [100]. Although such SiNPs with aptamers have excellent photostability and possess a great potential for separating and imaging both individual and multiple mixtures of cancer cells, they mostly rely on fluorescence-based methods that may suffer from inevitable autofluorescence in a real mixture or *in vivo*. To attain their full potential, further studies are necessary. Near-infrared dye or bioluminescence-based approaches are needed to expand the utility of SiNPs. In addition, the binding affinity of aptamer-conjugated SiNPs against living cells should be compared with that of other aptamer-NPs or antibody-conjugated SiNPs.

### 3.4. Aptamer-Conjugated Carbon Nanomaterials

Carbon nanomaterials, including graphenes and single-wall carbon nanotubes (SWCNTs), have attracted a lot of interest in the detection of cancer cells and bacteria [101–103]. One of the interesting properties of nanomaterials is their ability to act as a transducer or quencher with a tunable band gap and high elasticity [104–106]. Additionally, due to their hydrophobic surfaces, ssDNA molecules preferentially adsorb onto graphenes or SWCNTs by means of π-stacking interactions between the nucleotide bases and the sidewalls of the carbon nanomaterials [107].

In terms of the energy-absorbing ability, Wei *et al.* reported that graphene oxide (GO) could serve as an acceptor in the electrochemiluminescence (ECL) resonance energy transfer dubbed ERET and was designated to be a target recognition probe in combination with mucin 1 protein (MUC1) aptamer labeled with bis(2,2′-bipyridine)-(5-aminophenanthroline)ruthenium (II) (Ru1) (Apt-Ru1) [108]. Since Ru1 is a Ru(bpy)_3_^2+^ derivative, it possesses ECL behavior; efficient quenching of ECL occurred when the Apt-Ru1 bound with GO. However, in the presence of either MUC1 or MCF-7 cells, the ECL increased significantly due to the folded formation of the target-bound aptamer, which led to the release of the Ru1-aptamer from the surface of GO. Based on this strategy, they achieved a detection limit of 40 nM for purified MUC1 and 30 cells·mL^−1^ for MCF-7. Likewise, bacterial cells were measurable on the GO surface using a FAM (carboxyfluorescein)-aptamer which was specific for *S. typhimurium*. The increased fluorescence in the presence of the target bacteria was quantified as a function of bacterial cells, and the aptamer-based sensor reached an LOD as low as 100 CFU·mL^−1^ [101]. Cao *et al.* incorporated this FAM aptamer-immobilized GO (FAM-Apt-GO) into a 33-channel microfluidic chip for the sensitive detection of CCRF-CEM cancer cells [102]. The FRET between FAM-Apt and GO exhibited quenched fluorescence, whereas increased fluorescence intensity was observed when the target cells were present. The multichannel microfluidic chip based system was able to detect as few as 25 cells·mL^−1^ in a simultaneous and multiplexing manner.

Feng *et al.* reported a reusable graphene sensor functionalized with aptamer using electrochemical detection [109]. Briefly, the NH_2_-modified aptamer, specific to nucleolin of tumor cell, was conjugated with tetracarboxylic acid-functionalized graphene via a carbodiimide-mediated chemistry. The resulting graphene surface was used as a nanoscale anchorage substrate to effectively capture HeLa cells on the electrode. Based on the results of electrochemical impedance spectroscopy (EIS), which targeted the cells by monitoring the change in electron-transfer resistance (Ret) on the electrode, this method was able to detect low concentrations of HeLa cells with an LOD of 794 cells·mL^−1^. Furthermore, the strong association between the aptamer and the target cell was disrupted by the hybridization of the aptamer with its complementary DNA, which made this biosensor reusable after mild washing.

**Figure 6.**
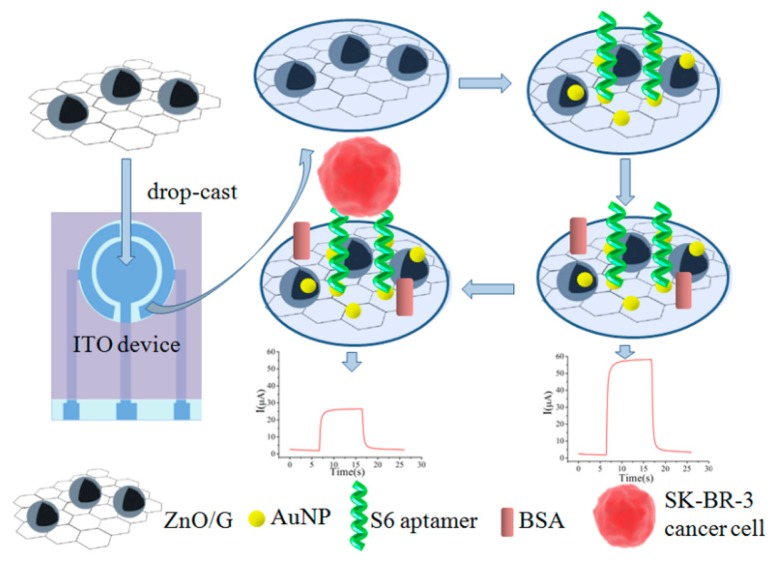
Schematic of the fabrication of an Apt-AuNP dotted ZnO/G modified indium tin oxide device. As shown, the ZnO/G composite was deposited onto the indium tin oxide micro device. Next, AuNPs were electrodeposited for the immobilization of aptamers specific for SK-BR-3 cancer cells. After the target cells are captured, photoelectrochemical measurements were carried out based on the addition of ascorbic acid. The image was adopted from [110].

Recently, Liu *et al.* demonstrated a ZnO/graphene (ZnO/G)-based Apt-AuNP composite for targeting SK-BR-3 cells on a portable indium tin oxide micro-device using photoelectrochemical detection (PEC) (Figure 6) [110]. In this assay, the AuNPs were electrodeposited onto the ZnO/G composite, followed by immobilization of the aptamer.

The PEC measurement of SK-BR-3 cancer cells captured by S6 aptamers was performed by the addition of ascorbic acid. As a result, the LOD was found to be 58 cells·mL^−1^. Wang and colleagues applied a similar approach to detect *Salmonella*. In this scenario, the GO and AuNPs were coated onto a glassy carbon electrode for EIS analysis [111]. In the presence of *Salmonella*, the aptamer immobilized on the AuNPs specifically captured the target, leading to retardation of electron transfer between the electrode and the electrolyte, producing a higher resistance. Using EIS, the detection sensitivity reached 3 CFU·mL^−1^.

For the synthesis of improved nano-composites, Yan *et al.* combined a porous GO/Au/aptamer composite with a thionine-functionalized porous PtFe alloy to target MCF-7 breast cancer cells [112], because the porous GO composites have several advantages compared to the conventional graphene, such as a large surface area, fast electron transportation, and good biocompatibility [113,114]. For the typical sandwich type assay, the GO/Au composite was deposited on a glassy carbon electrode, followed by conjugating the aptamer to recognize MUC1. Upon adding the nanoporous PtFe alloy conjugated with multiple anti-MUC1 aptamers, the final composite allowed for the sensitive detection of target MCF-7 cells with MUC1 overexpression. Using this amplification strategy, the detection limit was as low as 38 cells·mL^−1^.

**Figure 7.**
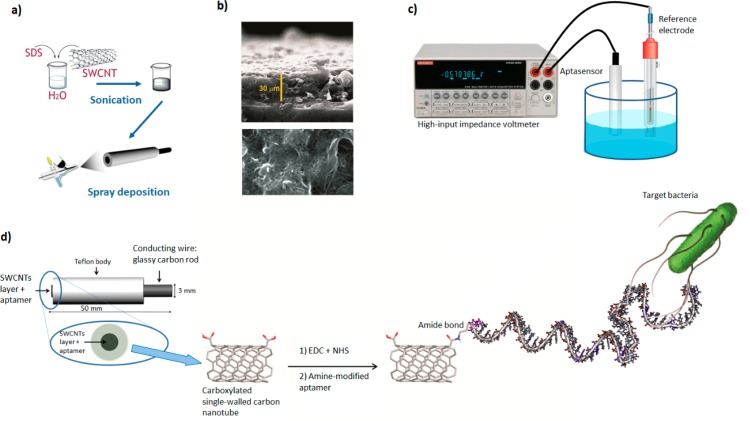
Schematic of the potentiometric-based detection of pathogenic bacteria using Apt-SWCNTs. (**A**) Deposition of SWCNTs on a glassy carbon electrode surface; (**B**) Environmental scanning electron microscope image of the deposited layer of SWCNTs on top of the glassy carbon electrode; (**C**) Experimental set-up for potentiometric measurements; (**D**) Over-view of the steps involved in the detection of bacteria using a potentiometric aptasensor. The image was adopted from [118].

Potentiometric analysis using SWCNTs as efficient ion-to-electron transduces was demonstrated for the ultra-sensitive detection [115]. Zelada-Guillen *et al.* reported an aptamer-conjugated SWCNT (Apt-SWCNT) for the potentiometric detection of pathogenic bacteria, where the Apt-SWCNT played dual roles in sensing and transducing (Figure 7) [116].

For the preparation of this device, a layer of SWCNTs was sprayed onto a glassy carbon rod that was electrically connected to a potentiometer and the NH_2_-modified aptamer against *S. typhimurium* was covalently conjugated with the SWCNTs to capture the target bacteria. While the aptamers were being self-assembled through π-π stacking interactions between the bases and the SWCNT walls, the binding of the target bacteria to the aptamers triggered conformational and charge changes in the aptamer, leading to a subsequent change in the potential. Such Apt-SWCNT-based methods are rapid and sensitive enough to detect 1 CFU·mL^−1^ of *S. typhimurium*. Additionally, the aptamer can be easily regenerated and reused after dissociating and reconditioning steps. Similar potentiometric methods based on Apt-SWCNT have been reported for the sensitive detection of various pathogenic bacteria including, *E. coli*, *S. aureus*, and *Salmonella paratyphi* A [117,118].

### 3.5. Aptamer-Conjugated Quantum Dots

Quantum dots (QDs) are semiconducting nanocrystals that exhibit exceptional optical and electrical behavior in cellular imaging and analysis [119–121]. Compared to conventional fluorophores, QDs have narrow and size-tunable emission spectra, high quantum yield, and great photostability [122,123]. Most importantly, since QDs exhibit multiplex emissions when excited with a single wavelength, QDs conjugated with aptamers (Apt-QDs) have been broadly used for the detection of various cancer cells and bacteria with high specificity, as well as being implemented for signal enhancement with high assay stability.

**Figure 8.**
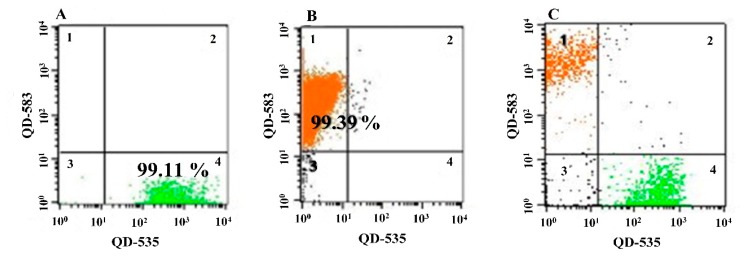
The flow cytometric analysis of either individual (**A**) *Vibrio parahaemolyticus*, (**B**) *S. typhimurium*; or (**C**) a mixture of both bacteria with Apt-QDs. The percentage of bacteria detected is also indicated in the figures. The image was adopted from [126].

It was reported by Lian *et al.* that poly(ethylene glycol)-phospholipid micelles in conjunction with CdSe/ZnS QDs modified with a thiol-ended aptamer, which specifically binds with nucleolin in cancer cell surface [124], were useful to specifically recognize breast cancer cells [125]. Lian *et al.* showed that the Apt-QDs successfully targeted MCF-7 and MDA-MB-231 cells with a strong red fluorescence compared to normal cells (NIH-3T3). Furthermore, the Apt-QDs exhibited no apparent cytotoxicity at the tested concentrations, indicating good biocompatibility and suitability for targeting cells. Similarly, Duan *et al.* used two QDs with different colors (green and orange) at a single excitation for the simultaneous detection of *Vibrio parahaemolyticus* and *S. typhimurium* [126]. After the QDs were conjugated with an aptamer specific for either *V. parahaemolyticus* or *S. typhimurium*, the Apt-QDs allowed for the individual or collective detection of two bacterial cells with a flow cytometer (Figure 8A–C). It was also reported that the cocktails of Apt-QDs, which contained three different types of aptamer-conjugated QDs specific to *E. coli* cells, were used to enhance the binding and detection sensitivity [127]. Compared to any individual aptamer or other combinations of aptamers, the cocktail mixture in the Apt-QDs exhibited an 18-fold higher LOD (371 CFU·mL^−1^). It should be noteworthy that aptamers evolved from cell-SELEX promote the binding affinity to target cells by recognizing multiple sites on the cell surface.

To further increase the number of QDs on the target, Hua *et al.* fabricated QD-decorated SiNPs (SiNP-QD) [128]. Along with two different aptamers targeting MUC1 or nucleolin in the tumor cell surface, they prepared two different sensing probes consisting of MUC1 aptamer-conjugated MBs and nucleolin aptamer-conjugated SiNP-QDs. Using two probes, the MCF-7 breast cancer cells were sensitively detected at a concentration as low as 85 cells·mL^−1^.

In addition to fluorescent detection based on QDs, Li *et al.* applied Apt-QDs to electrochemical stripping voltammetry for the detection of MCF-7 breast cancer cells [129]. After anti-MUC1 aptamers were hybridized with a complementary DNA anchored on the Au-electrode surface, the carboxyl QDs were conjugated with NH_2_-modified aptamers via EDC/NHS reaction, which was followed by the addition of MCF-7 breast cancer cells. Since the MUC1 on the MCF-7 cell surface could compete with cDNA, the Apt-QDs were released from the Au-electrode surface in the presence of the target cells. The number of QDs on the electrode was inversely proportional to the concentration of target cells, which was determined by the electrochemical stripping method. The competitive electrochemical cytosensor was able to detect concentrations as low as 100 MCF-7 cells·mL^−1^.

**Figure 9.**
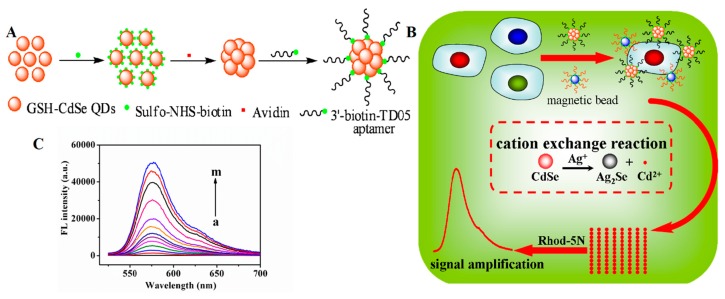
Schematic of the cation exchange based fluorescence method using QD nanoclusters. (**A**) Steps involved in the preparation of Apt-QD nanoclusters; (**B**) Overall schematic for the proposed cation exchange based fluorescence method. As shown, the cancer cells were targeted using both Apt-MBs and Apt-QD nanoclusters. The fluorescence intensity triggered by the cation exchange in QD NCs after the addition of Ag^+^ was measured for sensitive detection; (**C**) Fluorescent intensity of Rhod-5N dye by the cation exchange reaction in the presence of different numbers of Ramos cells. The image was adopted from [130].

A similar study was described by Sheng *et al.*, who used QD nanoclusters and MBs to develop a cation exchange reaction-based fluorescence method for the sensitive detection of Ramos cells (Figure 9) [130]. A biotinylated aptamer was conjugated with avidin-modified CdSe QDs and used for targeting cells. At the same time, a secondary aptamer that binds with a different site on the target cells was conjugated with MBs. The target cells were captured in a sandwich by the two probes (*i.e.*, Apt-QDs and Apt-MBs), allowing for magnetic separation. Upon the addition of Ag^+^ ions and the Rhod-5N dye (a nonfluorescent metal-sensitive dye), a large number of Cd^2+^ ions were released from the QD nanoclusters, resulting in fluorescence of the Rhod-5N dye. Under optimal conditions, the cation exchange-based method was able to detect concentrations as low as 50 cells·mL^−1^.

### 3.6. Aptamer-Incorporated DNA Nanostructures

Due to the ability of DNA to generate a myriad of folding structures, the aptamer-DNA conjugate is capable of carrying drug-like molecules to target cells by utilizing the target-specific aptamer and the drug-binding DNA structure [131,132]. It has been reported that two- and three-dimensional nanostructures based on the DNA origami provided distinct binding sites with various drug components [133,134]. The folding ability of DNA to produce many shapes is mainly relied on the Watson-Crick base-pairing between short nucleic acid sequences. Such a modified DNA nanostructure is easy controllable, biocompatible, and cell-permeable. In addition, the conjugation of aptamers to the DNA structures can improve the target specificity.

Chang *et al.* reported a distinct aptamer-DNA structure with icosahedral shape containing six-point-star motif, which was used as a nanocarrier with doxorubicin for cancer chemotherapy [135]. The DNA icosahedra was constructed using six individual DNA single strands, where five of the strands bended to a specific angle by hybridizing adjacent strands and the sixth strand was used for aptamer conjugation. For this, they employed a MUC1 DNA aptamer which specifically binds to MUC1 expressed in breast cancer cells (MCF-7) [136]. The doxorubicin was then intercalated into the aptamer-DNA icosahedral structure to construct Doxo-Apt-DNA-icosa. Under optimized conditions, the Doxo-Apt-DNA-icosa was not only internalized into MCF-7 cells but also showed a significantly higher cytotoxicity than free doxorubicin. This Apt-DNA-based nanostructure provides a controlled release of cancer drugs with site-specific targeting. Similarly, Zhu *et al.* demonstrated an aptamer-guided DNA nanotrain (Apt-NTr) for targeted transport of cancer drugs [137]. The aptamer in the Apt-NTr was screened from cell-SELELX using CEM cells [15] and was linked to two repeated hairpin DNA sequences at one end. This structure can be easily polymerized through Watson-Crick base-pairing. Furthermore, the Apt-NTr loaded with doxorubicin acts as an anticancer drug selectively to tumor CEM cells, whereas no cytotoxicity was observed for control cells [137].

Recently, Hu *et al.* reported an aptamer-conjugated FRET nanoflower (Apt-NF) which exhibited multi-fluorescence emissions by a single wavelength excitation [138]. Unlike the traditional Watson-Crick base-pairing, the multi-fluorescence NFs were achieved by incorporating three different dyes (FAM, Cy3 and ROX) into rolling circle replicon that can rapidly synthesize DNA. The template used for rolling circle replication consists of complementary aptamer toward CEM cells [15] and drug-loading sequence. The aptamer in NFs preserved its binding affinity and exhibited extremely bright fluorescence upon binding with CEM cells, but not with control Ramos cells. In addition to the imaging ability, the apt-NFs were also suitable for targeted drug delivery [138].

## 4. Conclusions

To generate high binding affinity without the knowledge of cell receptors, cell-SELEX is very useful for providing specific aptamers against various cancer cells and bacteria. This review shows that by utilizing cell-SELEX, recent advances in the development of aptamer-nano hybrid sensors have led to remarkable improvement in targeting cell. Since aptamers can be easily generated and modified with various nanomaterials, the traditional limits, related to low sensitivity, poor stability, and high cost can be overcome. Aptamer-nanomaterial hybrids show tremendous potential as robust diagnostic and therapeutic reagents for detecting and characterizing different types of cells. Given the complexity of cancer, the aptamers identified through cell-SELEX would be more advantageous than the conventional probes to simultaneously detect and differentiate normal and abnormal cells. Future efforts should focus on designing multimeric aptamers which will contribute to boosting the early detection of detrimental cells related to human diseases with high binding affinity and target specificity.

## Figures and Tables

**Table 1.** Summary of aptamers selected against cancer cells using cell-SELEX.

**Aptamer**	**K_d_^a^ (nM)**	**Target Cell**	**Control Cell Used for Negative Selection**	**Reference**
DNA	16.49 ± 0.40	gastric cancer cells, HGC-27	gastric epithelial cell, GES-1	[26]
	28.2 ± 5.5	non-small cell lung cancer (NSCLC)	HLAMP	[34]
	0.7 ± 0.2	colorectal cancer cells, (DLD-1)	HCT 116	[16]
	60 ± 8	H23 lung adenocarcinoma cells	HBE 135-E6/E7 normal epithelial lung cells	[28]
	0.76 ± 0.13	Ramos cells	-	[29]
	0.8	T-cell acute lymphoblastic leukemia (T-ALL) cells, CCRF-CEM	Ramos cells	[15]
	4.5 ± 1.6	acute myeloid leukemia (AML) cells (HL60)	NB4	[31]
	∼38	small-cell lung cancer (SCLC) cells, NCIH69	NSCLC cells, NCI-H661	[32]
	20 ± 10	human glioblastoma cells, U118-MG	human astroglial cells, SVGp12	[33]
	73.6 ± 11.01	PC-3 cells	RWPE-1, SMMC-7721 and Hela cells	[35]
	0.25 ± 0.08	TOV-21G	Hela cells	[36]
RNA	94.6	HER-2-overexpressing human breast cancer cells, SK-BR-3	HER-2 negative cells, (MDA-MB-231) and siHER-2-transfected SK-BR-3 cells	[30]
	1.5 ± 0.02	pancreatic cancer cell line (Mia-PaCa-2)	normal pancreatic cell line (HPDE)	[27]

a The K_d_ values obtained here correspond to the most potent binding aptamer.

**Table 2.** Summary of aptamers selected against bacteria using bacterium-SELEX.

**Aptamer**	**Target Bacteria**	**Control**	**K_d_^a^ (nM)**	**Reference**
DNA	*Mycobacterium tuberculosis*	*Mycobacterium bovis*	nd	[18]
	*Staphylococcus aureus*	*Streptococcus* and *S. epidermidis*	35	[37,44]
	*Shigella dysenteriae*	a mixture of related intact pathogenic bacteria	23.47 ± 2.48	[40]
	*Escherichia coli* O157:H7	*E. coli* K12 cells	∼110	[41]
	*E. coli*	a mixture of other bacterial species	12.4	[42]
	*Campylobacter jejuni*	non-*C. jejuni* strains	292.8 ± 53.1	[47]
	*Listeria monocytogenes*	a mixture of related intact pathogenic bacteria	48.74 ± 3.11	[49]
	*Pseudomonas aeruginosa*	*S. maltophilia* and *A. baumannii*	17.27 ± 5	[50]
	*Salmonella* O8	*E. coli* and *S. choleraesuis*	32.04	[45]
RNA and DNA	*Salmonella Enteritidis*	other Salmonella serovars	1.73 ± 0.54 μM	[38,46,48]

a The K_d_ values obtained here correspond to the most potent binding aptamer.
